# BanglaLekha-Isolated: A multi-purpose comprehensive dataset of Handwritten Bangla Isolated characters

**DOI:** 10.1016/j.dib.2017.03.035

**Published:** 2017-03-29

**Authors:** Mithun Biswas, Rafiqul Islam, Gautam Kumar Shom, Md. Shopon, Nabeel Mohammed, Sifat Momen, Anowarul Abedin

**Affiliations:** a Department of Computer Science and Engineering, University of Liberal Arts Bangladesh, Bangladesh; b Department of Computer Science and Engineering, University of Asia Pacific, Bangladesh

## Abstract

BanglaLekha-Isolated, a Bangla handwritten isolated character dataset is presented in this article. This dataset contains 84 different characters comprising of 50 Bangla basic characters, 10 Bangla numerals and 24 selected compound characters. 2000 handwriting samples for each of the 84 characters were collected, digitized and pre-processed. After discarding mistakes and scribbles, 1,66,105 handwritten character images were included in the final dataset. The dataset also includes labels indicating the age and the gender of the subjects from whom the samples were collected. This dataset could be used not only for optical handwriting recognition research but also to explore the influence of gender and age on handwriting. The dataset is publicly available at https://data.mendeley.com/datasets/hf6sf8zrkc/2.

**Specifications Table**

**Table:** 

Subject area	*Computer Science*
More specific subject area	*Image processing, Optical character recognition, Machine learning*
Type of data	*Images, Ms Excel*
How data was acquired	Subjects filled prearranged forms, which were then scanned.
Data format	*Processed and labeled*
Experimental factors	*Samples were collected scanned, processed, cropped and resized before presentation*
Experimental features	*None*
Data source location	*Dhaka and Comilla District of Bangladesh*
Data accessibility	*Data presented in this article is freely available from*https://data.mendeley.com/datasets/hf6sf8zrkc/2

**Value of the data**

• This data is very useful for training machine learning models [1] for optical handwriting recognition [2].
• This currently stands as the World׳s largest openly accessible dataset of Bangla handwritten characters. The number of samples makes it suitable for deep learning research [3].
• It is the only dataset (as of now) which comprises of Bangla basic characters, numerals and selected compound characters – all in one single place, unlike other existing data sets [4], [5].
• Apart from traditional handwriting recognition tasks, this dataset opens up opportunities for novel research along the line of judging the aesthetic quality of handwriting.
• The dataset can be used to potentially explore patterns related to age and gender in handwriting samples.

## 1 Data

The data contains handwriting samples of all Bangla basic characters and numerals (i.e. 50 basic characters and 10 numerals). Furthermore, it also contains 24 selected compound characters. Thus the dataset contains a total of 84 different Bangla characters. After collecting the raw data on forms, the samples are digitized, pre-processed and stored in publicly accessible location. The collected samples are from subjects of different age groups ranging between 6 and 28. After omitting clear mistakes and scribbles, the dataset contains a total of 1,66,105 digitized images of Bangla characters.

## 2 Experimental design, materials and methods

### 2.1 Data collection

While there has been a lot of success in the automatic recognition of handwritten English content [6], the state of automatic Bangla handwriting recognition research is lagging far behind. Recent trends have shown that machine learning, specifically deep learning techniques, can be very effective in tackling handwriting recognition tasks. However, such learning mechanisms usually require large quantities of labelled data. BanglaLekha-Isolated, the dataset presented in this paper, aims to provide such a collection.

**Fig. 1:**
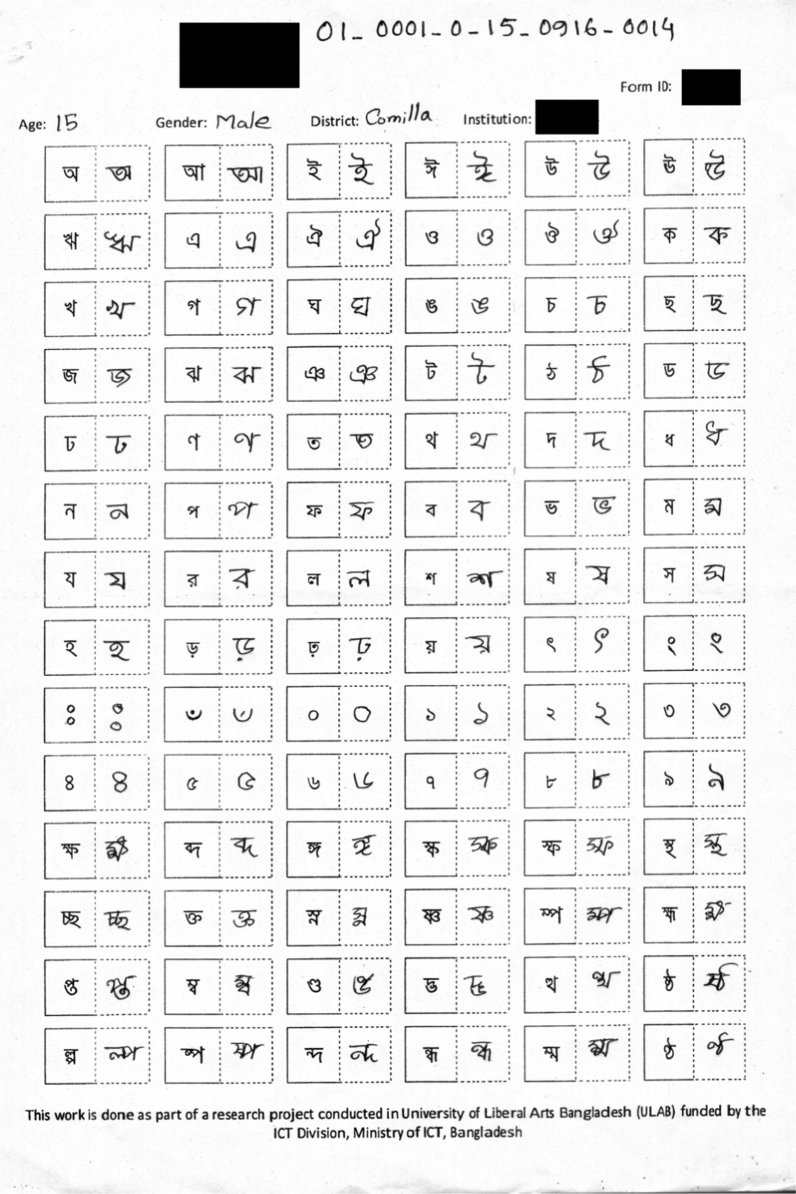
Example of a filled data collection form.

**Fig. 2:**
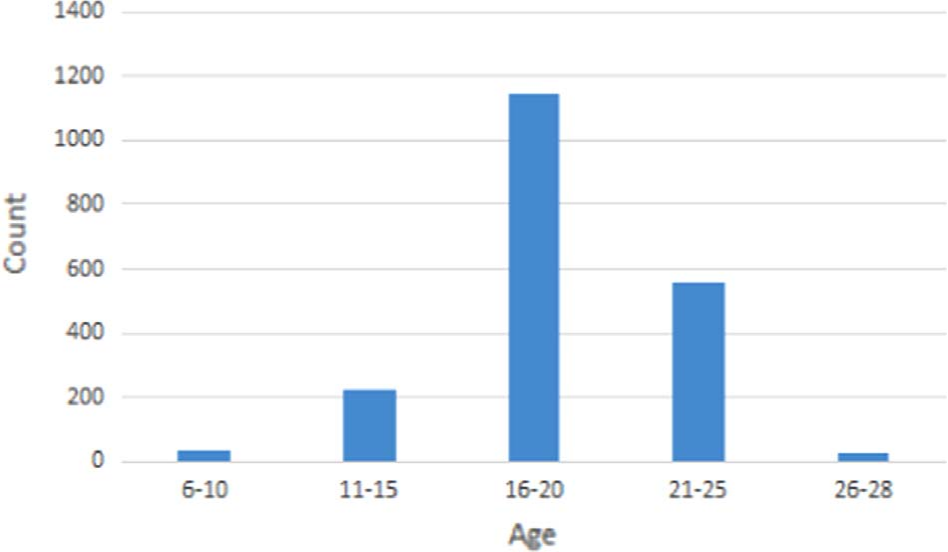
Age distribution of the subjects.

This dataset concentrates exclusively on isolated characters. Fig. 1 shows a sample of the form used to collect handwriting samples. Subjects were asked to fill in such forms at their own pace, then within 5 min and then within 2 min. This was done to get a good distribution of handwriting quality. The gender and age of each subject was also recorded. The age group of the subjects ranged from 6 years to 28 years with a high density between the ages of 16–20 (Fig. 2). 59.4% of the subjects were males while the remaining 40.6% were females.

### 2.2 Data processing

Each form was given a unique 17 digit identification number. The identification number is given as follows: the first two digit identifies the district the participant lives in. Currently, there are only two districts: Dhaka and Comilla. The next four digits identify the institution of the subject. Afterward, a single digit is used to identify the gender of the participant (0 – male, 1 – female). The following two digits captures the age and the next four digits the date on which the form was filled up. The last four digits of the form identification number is a basic serial number of the form. Each part of the identifier is separated by an underscore (_). This makes the identification number of the form 22 characters long.

**Fig. 3:**
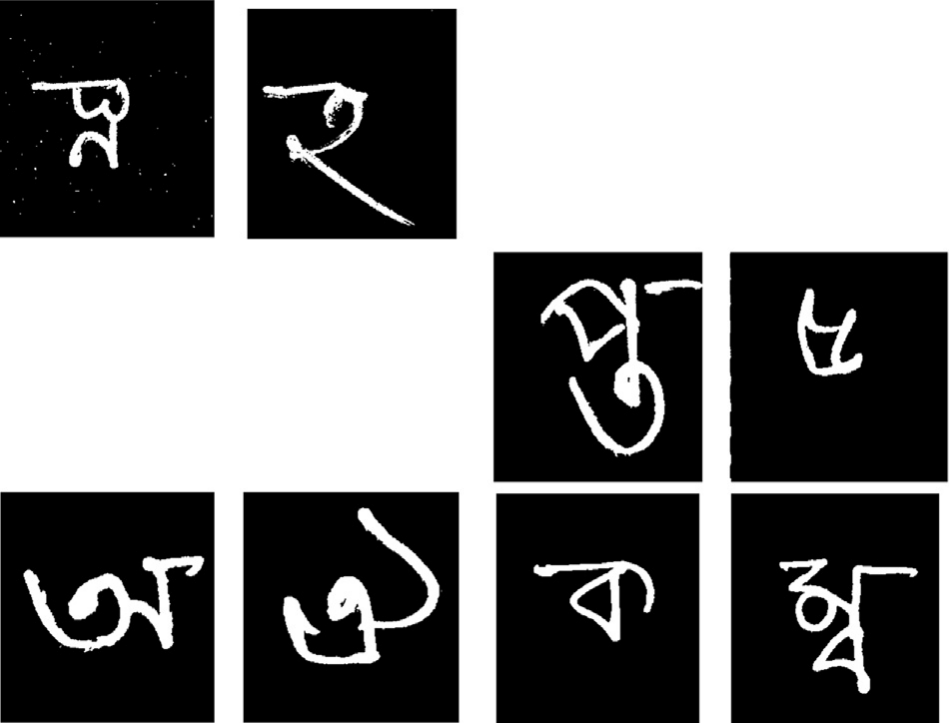
Sample images from the dataset.

The forms were then scanned (at 600 dpi), and each handwritten character was extracted automatically and the extraction was verified manually. In the original scanned versions, the background is white and the writing appears in black. As the dataset is envisioned to be used for machine learning/pattern recognition tasks, the background was converted to black and the characters samples were converted to white. A median filter was employed to reduce image noise, which was followed by the application of an edge thickening operation to bring clarity to the images. Fig. 3 shows example pre-processed images of the dataset.

Each of the 84 characters in the form were numbered (from 1 to 84). The final file name of each image was a concatenation of the form identification number followed by the number to which the concerned character was mapped. Once again an underscore (_) separates the two parts. Thus, the filenames can be used to infer the character whose image the file contains as well as the age and gender of the person who wrote it in the form. For convenience, the image files have been organized by character in folders, i.e. 84 folders, one folder per character.

### 2.3 Data on the aesthetic quality of handwriting

Apart from the information gathered from the forms (age, gender and the actual character), the dataset also provides a spreadsheet which contains marks given to individual forms (group of 84 characters) as a judgment of the aesthetic quality of the letters (how beautiful is the handwriting?). While marking the characters, the following criteria were set by a nationally (in Bangladesh) recognized handwriting expert [7].

a) Consistent Size and format
b) Clear and easy to read
c) One style throughout the form
d) Proper dimension
e) Correctness.

This criteria was then used by three assessors, each of whom are literate native Bangla speakers. Each assessor assessed each form independently, and awarded a mark between 0 and 5, where 0 means poor and 5 means excellent. The marks awarded to each form by each assessor is included in a spreadsheet, which is also openly available.
